# Evaluation of a point-of-care tuberculosis test-and-treat algorithm on early mortality in people with HIV accessing antiretroviral therapy (TB Fast Track study): study protocol for a cluster randomised controlled trial

**DOI:** 10.1186/s13063-015-0650-0

**Published:** 2015-03-28

**Authors:** Katherine L Fielding, Salome Charalambous, Christopher J Hoffmann, Suzanne Johnson, Mpho Tlali, Susan E Dorman, Anna Vassall, Gavin J Churchyard, Alison D Grant

**Affiliations:** Department of Infectious Disease Epidemiology, London School of Hygiene & Tropical Medicine, Keppel Street, London, WC1E 7HT UK; Aurum Institute, 29 Queens Road, Johannesburg, 2041 South Africa; School of Medicine, Johns Hopkins University, 1503 E. Jefferson Street, Baltimore, Maryland 21231 USA; Technical Assistance Cluster, Foundation for Professional Development, 173 Mary Road, Pretoria, 0184 South Africa; Department of Global Health and Development, London School of Hygiene & Tropical Medicine, Keppel Street, London, WC1E 7HT UK; Department of Clinical Research, London School of Hygiene & Tropical Medicine, Keppel Street, London, WC1E 7HT UK

**Keywords:** Tuberculosis, HIV infections, Pragmatic clinic trials, Mortality, Treatment

## Abstract

**Background:**

Early mortality for HIV-positive people starting antiretroviral therapy (ART) remains high in resource-limited settings, with tuberculosis the most important cause. Existing rapid diagnostic tests for tuberculosis lack sensitivity among HIV-positive people, and consequently, tuberculosis treatment is either delayed or started empirically (without bacteriological confirmation). We developed a management algorithm for ambulatory HIV-positive people, based on body mass index and point-of-care tests for haemoglobin and urine lipoarabinomannan (LAM), to identify those at high risk of tuberculosis and mortality. We designed a clinical trial to test whether implementation of this algorithm reduces six-month mortality among HIV-positive people with advanced immunosuppression.

**Methods/design:**

The TB Fast Track study is an open, pragmatic, cluster randomised superiority trial, with 24 primary health clinics randomised to implement the intervention or standard of care. Adults (aged ≥18 years) with a CD4 count of 150 cells/μL or less, who have not received any tuberculosis treatment in the last three months, or ART in the last six months, are eligible. In intervention clinics, the study algorithm is used to classify individuals as at high, medium or low probability of tuberculosis. Those classified as high probability start tuberculosis treatment immediately, followed by ART after two weeks. Medium-probability patients follow the South African guidelines for test-negative tuberculosis and are reviewed within a week, to be re-categorised as low or high probability. Low-probability patients start ART as soon as possible. The primary outcome is all-cause mortality at six months. Secondary outcomes include severe morbidity, time to ART start and cost-effectiveness.

**Discussion:**

This trial will test whether a primary care-friendly management algorithm will enable nurses to identify HIV-positive patients at the highest risk of tuberculosis, to facilitate prompt treatment and reduce early mortality. There remains an urgent need for better diagnostic tests for tuberculosis, especially for people with advanced HIV disease, which may render empirical treatment unnecessary.

**Trial registration:**

This trial was registered with Current Controlled Trials (identifier: ISRCTN35344604) on 12 September 2012.

## Background

### Background and rationale

Early mortality among HIV-positive people starting antiretroviral therapy (ART) remains higher in resource-constrained settings compared with industrialised countries [1]. Tuberculosis is the most important cause of death among people with HIV worldwide [2], and limited data suggest that this remains true of people starting ART [3]. However, the diagnosis of tuberculosis is difficult and may be missed altogether, particularly among people with low CD4 counts, who are less likely to be diagnosed using sputum-based tests because of lower mycobacterial concentration or inability to produce sputum, and may have atypical appearances on chest radiography. Sputum mycobacterial culture is the gold standard diagnostic test for tuberculosis, but is not routinely available in many resource-constrained settings. Where culture is available, the result may take up to six weeks, which may delay ART initiation [4].

New diagnostic tests for tuberculosis are increasingly available. In South Africa, Xpert MTB/RIF has replaced sputum microscopy as the first diagnostic test for tuberculosis [5]. With an instrument turnaround time of under two hours, Xpert MTB/RIF has the potential to provide point-of-care testing with in-session results, but the instrument requirements, its moderate complexity [6,7] and high cost mean that it is unlikely to be widely implemented at primary care level in resource-constrained settings. Xpert MTB/RIF has better sensitivity than microscopy, but is less sensitive than sputum culture on liquid media [8]. Further, in two trials comparing Xpert MTB/RIF to sputum smear microscopy, Xpert did not improve patient-relevant outcomes. In the XTEND trial, nested within national roll-out of Xpert MTB/RIF in South Africa, placed in off-site laboratories, there was no reduction in mortality among people being investigated for tuberculosis [9]. In the TB-NEAT trial Xpert MTB/RIF was deployed in primary care clinics to give a same-day result, with no resulting reduction in morbidity [10]. In both trials, a likely explanation was that many people were treated empirically (that is, without microbiological confirmation) for tuberculosis, and that Xpert MTB/RIF provided microbiological confirmation for some cases which would otherwise have been treated empirically, but found few additional cases.

Empirical treatment of tuberculosis is common, but often requires a physician’s decision. Many primary care clinics are run by nurses with physicians attending only occasionally, if at all, and in such settings empirical treatment may be delayed, or not initiated. We sought to develop an algorithm which would enable nurses in primary care settings to identify, among HIV-positive people with advanced immunosuppression who are at high risk of death, those at the highest risk of tuberculosis, in order to start empirical tuberculosis treatment promptly, followed by ART.

A promising candidate component of this algorithm was an assay for lipoarabinomannan (LAM), a mycobacterial cell-wall component which can be detected in urine among patients with tuberculosis. A lateral flow assay for LAM is commercially available as a point-of-care test, giving a result in 25 minutes at relatively low cost which, with no requirement for sample processing, has potential for use in primary care clinics. The test’s sensitivity is too low to be useful for HIV-negative people. Even among HIV-positive people, sensitivity is inadequate, except among those with very low CD4 counts [11]. We therefore selected two additional markers for our algorithm: body mass index (BMI) and haemoglobin. Among people with HIV, low BMI and low haemoglobin levels are strongly associated with early mortality, and also with active tuberculosis [12-17]. Both BMI and haemoglobin can be measured by a nurse in a primary care setting with in-session results. Additional analysis of data from a study evaluating LAM among hospitalised patients in South Africa with signs or symptoms of tuberculosis [18] helped refine the algorithm.

**Figure 1:**
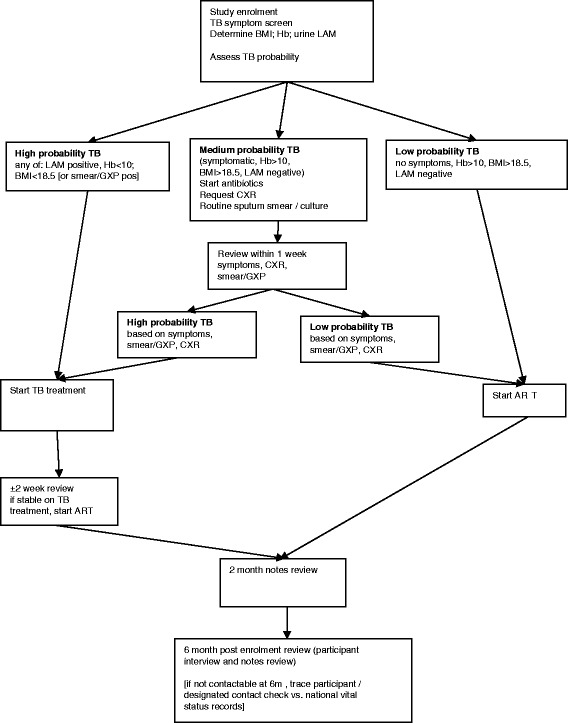
**TB Fast Track management algorithm.** ART: antiretroviral therapy; BMI: body mass index; CXR: chest radiograph; GXP: Xpert MTB/RIF; Hb: haemoglobin; LAM: lipoarabinomannan (urine assay for tuberculosis); TB: tuberculosis.

We therefore decided to evaluate an algorithm combining haemoglobin, BMI and urine LAM lateral flow assay with symptom screening for tuberculosis, to classify study patients as having high, medium or low probability of active tuberculosis (Figure 1), and accordingly start presumptive tuberculosis treatment, ART or both, with minimal delay.

### Rationale for a randomised controlled trial

Our overall aim was to enable the rapid initiation of tuberculosis treatment among high-risk people, and thus reduce early mortality due to tuberculosis. Rapid initiation of tuberculosis treatment should also facilitate early initiation of ART, thus reducing early mortality due to a wide range of conditions associated with advanced HIV disease. Other potential advantages include a lower risk of morbidity associated with immune reconstitution syndrome due to tuberculosis, reduced morbidity due to rifampicin-sensitive bacterial infections and effective treatment for latent tuberculosis infection.

However, a strategy promoting rapid initiation of tuberculosis treatment without bacteriological confirmation has potential disadvantages. For patients, some will receive tuberculosis treatment when they do not, in reality, have active tuberculosis, with attendant risks of adverse effects and drug interactions. Some patients with unidentified drug-resistant tuberculosis may be inappropriately treated with a regimen appropriate only for drug-sensitive tuberculosis. The focus of the algorithm on tuberculosis could also delay investigation and treatment for alternative co-morbidities. For the health system, disadvantages include a higher caseload of patients requiring tuberculosis treatment, and an apparent increase in tuberculosis case notification rates. Given uncertainties concerning whether the expected benefits of our novel management algorithm would outweigh the risks, we propose to conduct a clinical trial.

### Rationale for a cluster randomised trial

This trial could broadly be considered a health service delivery trial; for such trials, there is debate about whether randomisation should be at the individual or cluster level [19,20]. Advantages of cluster randomisation include minimising contamination, in that where patients in the same clinic are randomised to different arms, elements of the intervention may be implemented among patients allocated to the control arm. Other advantages include logistical convenience and approximating more closely how the intervention would be delivered in routine practice. Disadvantages include a larger sample size for the same power and effect size, complexities of revising sample size calculations if necessary, challenges in controlling for confounding and standardising delivery of the intervention. We decided on randomisation at clinic level primarily because we anticipated that contamination would be likely if individuals in one clinic could be randomised to either intervention or control arm.

### Hypothesis

We hypothesise that, for adults with HIV with a CD4 count of 150 cells/μL or less who are not taking ART, a care pathway using point-of-care tests to rapidly identify individuals at high risk of tuberculosis and ensure initiation of tuberculosis treatment followed by ART, will reduce mortality within the six months following enrolment.

## Methods/design

### Study design

The study is an open, pragmatic two-arm cluster randomised superiority trial, with primary health clinics as the unit of randomisation. The intervention is integrated into routine clinic activities as far as possible: study staff implement the algorithm, and all other aspects of care for the patient are delivered by clinic staff.

### Intervention arm - implementation of the algorithm

The study nurse completes a questionnaire with consenting, eligible patients to determine tuberculosis symptoms and BMI, and performs point-of-care tests for haemoglobin concentration and urine LAM (Determine™ TB LAM Ag, Alere, Waltham Massachusetts, United States). Patients are then classified as having high, medium or low probability of active tuberculosis (Figure 1).

Participants are classified as at a high probability of tuberculosis if they have: (i) a positive urine LAM result (defined as any positive band on the test strip, graded against the manufacturer’s pre-January 2014 reference card), (ii) a haemoglobin concentration <10 g/dL or (iii) a BMI <18.5 kg/m^2^ (along with any patient with a previous sputum smear or positive Xpert MTB/RIF test [Cepheid, Sunnyvale, CA, USA] result). They are started on tuberculosis treatment immediately, followed by ART as soon as possible.

Participants classified as at a low probability of tuberculosis, defined as no symptoms suggestive of tuberculosis, a negative LAM result, a haemoglobin concentration ≥10 g/dL and a BMI ≥18.5 kg/m^2^ (and not known to be sputum smear or Xpert MTB/RIF positive) are started on ART (without tuberculosis treatment) as soon as possible.

Participants classified as at a medium probability of tuberculosis, that is, reporting any symptom suggestive tuberculosis but not satisfying the criteria for high probability, are managed in accordance with national guidelines for test-negative tuberculosis: given broad spectrum (non-quinolone) antibiotics if indicated, an additional routine sputum sample is taken for smear and mycobacterial culture in line with routine practice, and a chest radiography is arranged if not recently performed, with review within one week wherever possible. At review, they are classified as high or low probability, based on symptom review, response to antibiotic treatment and investigation results.

Patients with specific symptoms suggesting diagnoses other than tuberculosis are managed in accordance with national guidelines. For study participants who start tuberculosis treatment, an efavirenz-based ART regimen is used, to be compatible with tuberculosis treatment. Any patients unable or unwilling to take this ART regimen are excluded from the trial. Outside this situation, patients can take any ART regimen consistent with South African ART guidelines.

In order to have a reference ‘gold standard’ against which to assess the study algorithm, a sputum specimen is also collected at enrolment for smear, culture, organism identification and drug susceptibly testing at a research laboratory. Results are not available at the time of enrolment, and so do not contribute to the initial assignment of probability of tuberculosis.

### Standard of care

Study staff recruit eligible, consenting patients and complete a short questionnaire, measure height and weight (for systematic measurement of BMI for analysis purposes). Further assessment for tuberculosis and preparation for ART is undertaken by clinic staff according to routine practice following national guidelines. In both arms of the study, dried blood spots and a urine sample are collected and stored. Intended tests include cryptococcal antigen and culture of urine for mycobacteria.

### Selection of study clinics

The study is conducted in primary care clinics and community health centres in a mixture of urban and rural settings in Gauteng, North West and Limpopo provinces, South Africa. Exclusion criteria include having a laboratory doing sputum smear microscopy and Xpert MTB/RIF tests onsite, with same day results. There was a deliberate decision to include rural sites on the basis that the intervention is particularly relevant to patients in rural settings, where long distances to clinics may be a barrier to speedy initiation of ART, particularly if multiple visits are needed.

### Study population

The study population are HIV-positive adults (≥18 years) with a CD4 count of 150 cells/μL or less, who are willing to start ART. The trial aims to be as generalisable as possible so exclusion criteria are few and include, among others, patients currently on tuberculosis treatment, who have completed tuberculosis treatment in last three months or ART in the last six months, those with a contraindication to efavirenz and those who are too sick to be managed in ambulatory care. We chose a CD4 count threshold of 150 cells/μL or less primarily because of the high risk of mortality in this group. A more standard CD4 count cutoff of under 200 cells/μL would have made the study more widely applicable, though at a cost of having a larger sample size due to a lower mortality rate among ART initiators with a CD4 count between 150 and 200 cells/μL.

### Trial outcomes

The primary outcome is all-cause mortality measured over a six-month period following enrolment. Secondary outcomes are (i) duration of hospital admission in the first six months after enrolment, (ii) time from enrolment to ART start, (iii) proportion of patients retained in HIV care and (iv) serious and severe adverse events in specified categories as defined by the study (particularly hepatotoxicity, hypersensitivity, peripheral neuropathy and nephrotoxicity). The study has also defined economic outcomes relating to measuring cost-effectiveness which include total diagnostic cost of the study cohort in intervention and control arms, as well as incremental diagnostic, treatment and per disability-adjusted life year (DALY) costs of the intervention compared to control from a provider perspective, over the six-month follow-up period.

Vital status at six months from enrolment will be ascertained from patient interview and medical records, and supplemented with information from the participant’s nominated next of kin for those lost to follow-up, and the South African mortality registration for participants with a South African identification number. If necessary, a trial endpoints committee will be convened, masked to study arm, to assign death endpoints where there are discrepant dates of death. Secondary outcomes will be measured using a combination of data from patient interview and medical records, abstracted at two and six months from enrolment. A substudy among study participants who die is being conducted, using needle and verbal autopsy methods to ascertain causes of deaths.

### Economic evaluation

An economic evaluation is being conducted from a provider perspective. The primary outcome for the economic evaluation is incremental cost per death averted within the trial period. An additional analysis will be conducted to estimate incremental cost per DALY averted. The costs of the intervention are being collected throughout the trial period, with care taken to exclude any research-related costs. A bottom-up costing approach will be used to measure the costs associated with implementation of the point-of-care tests at the clinic level (cost per person assessed and costs for different components of the algorithm). Provider costs for all services in the patient pathway are being collected from a sample of six clinics, purposively selected based on the ratio between staffing levels and the number of clinic attendees. Patient-level data on health service use are being collected using the general trial instruments (follow-up visits) and case note data. Should the intervention be found cost-effective, a budget impact analysis will be conducted to estimate the costs of national scale-up.

### Sample size considerations

Data from the TB and HIV Prevention, Care and Treatment programme of The Aurum Institute helped inform the sample size calculation for the primary outcome. Mortality in the first six months after ART initiation among those with a CD4 count of under 150 cells/μL was 24/100 person-years [17,21,22]. For the sample size calculation, this was considered a minimum estimate, given that ascertainment of deaths did not include a search of national death registrations for some of these studies, and mortality prior to ART exceeds mortality during ART. Given our study is designed to enrol patients prior to ART start we used a mortality estimate of 25/100 person-years in the standard of care arm. The sample size calculation took into account the clustered design [19] through the coefficient of variation, which we assumed to be between 0.2 and 0.25. We had no pre-study clinic-level mortality data to inform coefficient of variation between clinics, but given the study population were patients pre-ART, all with CD4 counts of 150 cells/μL or less, we assumed a coefficient of variation no greater than 0.25 was reasonable.

Tuberculosis is identified at autopsy in 21 to 54% of HIV-positive adults in sub-Saharan Africa [2]. Given the very high prevalence of tuberculosis in South Africa [3], we assumed that 60% of early deaths in our study population would be attributable to tuberculosis, and that, through earlier initiation of tuberculosis treatment, 66% of these could be averted. Using data from the TB and HIV Prevention, Care and Treatment programme of The Aurum Institute, among individuals with a CD4 count between 100 and 150 cells/μL, a two-month delay in ART initiation resulted in a 150% increase in mortality by six months; from 7% with immediate ART initiation to 18% if there was a two-month delay [22]. There must be some overlap of preventable deaths between these two effects, which we could not quantify precisely. Overall, we assumed that the intervention had the potential to result in a 40% reduction in mortality by six months after study enrolment.

Assuming 10 clinics per arm, 175 patients per clinic, 5% individuals whose vital status could not be ascertained at six months, estimated mortality of 25/100 person-years in the standard of care arm and coefficients of variation of 0.2 and 0.25, there would be 91% and 85% power to assess a 40% reduction in mortality, respectively. If the coefficient of variation is 0.2, there would be 81% power to assess a 35% reduction in mortality.

After enrolling participants for six months we established that for some clinics, the target of 175 patients per clinic was unlikely to be achieved, and so the sample size calculations were revised. Assuming a harmonic mean of 109 patients per clinic, by randomising an additional four clinics, the study maintained similar power and effect sizes as with the original calculation.

### Randomisation

The initial randomisation of 20 clinics was based on restriction, common in cluster randomised trials with a small number of clusters [19], and based on having reasonable balance, separately, for mean CD4 count, peri-urban/rural location of the clinic and the total number of new ART initiations per month. Using these criteria, 11,160 (6.0%) of the 184,756 possible allocations for randomising 20 clinics to two arms (ratio of 1:1) were identified. The validity of this restriction was checked for each allocation by comparing the number of times a cluster appeared in the same arm with each other cluster. There was no extreme imbalance and this restriction was assumed to be valid. The randomisation was organised by a statistician. In a public ceremony with study clinics represented by clinic managers and other HIV and TB programme staff, one allocation was chosen at random. Due to the revision in sample size, a second public randomisation took place 14 months later, where four additional clinics were randomised in a ratio 1:1 to the intervention or standard of care arm.

### Data collection and management

Case report forms were piloted in 10 clinics prior to the study start. Before a clinic formally initiated enrolment, the enrolment, one-week and two-week case report forms were tested in around 10 patients.

Written informed consent is obtained from all participants using information sheets available in relevant languages, with the assistance of a translator where necessary, using standard consent forms. Participants unable to read or write are asked to make a mark or thumbprint in the presence of a witness. Following enrolment, participants in the two arms are seen in accordance with the visit schedule summarised in Table 1. Study staff complete case report forms on carbonated paper in duplicate, and store forms at site in locked filing cabinets, with access to these records restricted to specified study team members. Case report forms are identified using the participant’s study number only, with locator information stored separately.

**Table 1:** **Summary of study procedures**

		**Intervention**						**Control**						
	**Enrolment**	**1 week (or less)**	**2 weeks**	**1 month**	**2 months**	**4 months**	**6 months**	**Enrolment**	**1 week (or less)**	**2 weeks**	**1 month**	**2 months**	**4 months**	**6 months**
Informed consent	√							√						
Locator information	√							√						
Baseline questionnaire	√							√						
TB symptom screen	√	√^a^						√						
Body mass index	√							√						
Lab tests														
Haemoglobin	√													
Urine lipoarabinomannan	√	√ ^a^												
Sputum TB microscopy and culture	√													
Urine/dried blood spot for storage	√							√						
TB patient follow-up visit			√^b^											
Patient record review		√			√		√		√			√		√
6 month questionnaire^c^							√							√
Patient contact calls		√		√	√	√	√		√		√	√	√	√

^a^For those at medium probability of TB according to the study algorithm. ^b^For those started on TB treatment, to check if stable on TB treatment and ready to start antiretroviral therapy. ^c^A subset of participants also have this questionnaire repeated at 12 months from enrolment. TB, tuberculosis.

Participant retention is crucial for this study, in particular for the measurement of the primary outcome. At enrolment in both arms, participants are asked to give locator information, including next of kin and their South African identification number, as described previously. Participants are also asked to provide their mobile telephone number, and are called by study staff at one week, and one, two, four and six months to reconfirm contact details. Participants are encouraged to notify study staff of any relevant changes in their circumstances, such as moving away from the locality.

A quality control system is in place to monitor 100% of the consent forms and enrolment case report forms, and 10% of follow-up case report forms. More frequent monitoring of a site is initiated if problems are identified. Every week, across all 24 sites, the bottom copies of completed forms are sent to a central office for data entry in a password-protected, user-limited sequel database, with range checks on fields where appropriate. Queries based on data in the database are generated every one to two months and sent to sites for resolution. Data in the database from all case report forms from a random sample of 10% of study participants are verified. In addition, data on critical fields, primarily used for measurement of study endpoints, are verified in the database for 100% of study participants. Locator data are stored separately from the main database.

### Statistical analysis

Analyses will use methods appropriate for the cluster randomised trial design, giving each cluster equal weight. Quantitative outcomes will be summarised as the mean for each cluster and the difference of means for the intervention versus standard of care arm. For binary outcomes or rate outcomes, the overall risk (rate) for each cluster will be calculated, as well as the ratio for the intervention versus standard of care arm. Given the overall randomisation was conducted using two strata (20 and four clinics in each stratum), the standard error for each effect measure will take into account stratified randomisation. An adjusted analysis will be conducted if, after visual inspection, imbalance by study arm in patient- or clinic-level factors is observed, to help reduce variability across clinics with respect to primary and secondary outcomes.

Subgroup analyses will be conducted for the primary outcome for baseline CD4 count (<50 or ≥50 cells/μL), previous tuberculosis history (from self-report; no previous or previous tuberculosis), baseline BMI (<18.5 or ≥18.5 kg/m^2^) and baseline haemoglobin (<8 or ≥8 g/dL). A statistical analysis plan documents the analysis of all trial outcomes.

### Ethics and dissemination

The trial has approval from the Research Ethics Committees of the University of Witwatersrand (approval number: R14/49 M111177); the London School of Hygiene and Tropical Medicine, United Kingdom (approval number: 6099) and the Provincial Research Committees of Gauteng, North West and Limpopo. It is registered with the South African Medicines Control Council as a phase 4 clinical trial (identifier: N2/19/8/2 () #20120157), and with Current Controlled Trials (identifier: ISRCTN35344604 [23]) and the South African registry (identifier: DOH-27-0812-3902).

The trial results will be communicated to stakeholders through dissemination meetings and to trial participants using language-appropriate information sheets. Investigators will present results at relevant conferences, and submit manuscript(s) to peer-reviewed journals. Public access to the participant-level dataset of main trial results and statistical code will be made available.

### Trial governance

The trial is governed by the Trial Steering Committee (TSC) and the Data Monitoring Committee (DMC). The TSC oversees the trial, monitors its progress and receives reports from the DMC, and advises the Chief Investigator and investigator team. The DMC’s role is to protect and serve trial patients, to assist and advise the Chief Investigator and the TSC so as to protect the validity and credibility of the trial and monitor the overall conduct of the clinical trial. The DMC meets approximately every six months, followed by a TSC meeting. Protocol amendments are submitted to all ethics committees and communicated to all investigators, the TSC and DMC.

## Discussion

This study aims to evaluate whether early mortality can be reduced, among ambulatory people with advanced HIV disease not taking ART, by an algorithm which enables nurses in primary care clinics to identify patients at highest risk and rapidly start them on tuberculosis treatment. A number of other recent trials, summarised in Table 2, aim to address the problem of high early mortality among people with advanced HIV disease entering care. The PrOMPT [24] and REMEMBER [25] studies aimed to evaluate empirical tuberculosis treatment among people with HIV and very low CD4 counts who screened negative for active tuberculosis at study entry. Recruitment to the PrOMPT study was much slower than anticipated and as a result the study was discontinued. The REMEMBER trial, with sites in Brazil, Haiti, India, Peru and five African countries, has a primary outcome of mortality at 24 weeks. Recruitment is complete and results are expected in 2015. The STATIS trial compares, among people with no overt evidence of tuberculosis at study entry, empirical tuberculosis treatment to intensive investigation for tuberculosis among HIV-positive patients with a CD4 count under 100 cells/μL in Cambodia, Côte d’Ivoire, Uganda and Vietnam [26]. Intensive investigation comprises Xpert MTB/RIF, urine LAM tests and a chest radiograph at baseline and at every follow-up visit, with tuberculosis treatment guided by these investigations. The primary outcome is death or invasive bacterial infection by 24 weeks.

**Table 2:** **Summary of trials of interventions to reduce early mortality among HIV-positive people starting antiretroviral therapy**

**Study**	**Setting**	**Randomisation level**	**Study population (main criteria)**	**Intervention**	**Outcome(s)**	**Expected date of results**
PrOMPT^a^[24]	Gabon, Mozambique, South Africa, Uganda	Individual	CD4 count <50 and body mass index <18; no previous TB treatment; aged ≥18 years; Sputum smear negative; and not fulfilling World Health Organization criteria for smear-negative TB.	4-drug TB treatment, followed by ART within 2 weeks. Comparator: ART alone	Primary: all-cause mortality in the first 24 weeks after initiation of ART, CD4 cell increase, safety, HIV viral suppression, TB incidence after ART initiation	N/A
REMEMBER [25]	Brazil, Haiti, India, Kenya, Malawi, Peru, South Africa, Tanzania, Zimbabwe	Individual	CD4 count <50; aged ≥13 years; Karnofsky ≥30; no previous TB treatment (within 96 weeks). Those with confirmed or probable TB excluded.	ART initiation within 3 days and 4-drug TB treatment within 7 days of ART start. Comparator: ART initiation within 3 days	Primary: survival at 24 weeks; survival over 96 weeks; time to AIDS progression; AIDS-free survival at 24 and 28 weeks; HIV viral load at 2, 24 and 48 weeks; safety	May 2016
REMSTART [27]	Tanzania, Zambia	Individual	Initially CD4 count <100, broadened to <200 following slow enrolment; aged ≥18 years. All screened for TB at enrolment with Xpert MTB/RIF	Rapid initiation of ART, screening for cryptococcal antigen, weekly home visits for 4 weeks by lay workers and rescreening for TB using Xpert MTB/RIF at 6 weeks. Comparator: standard of care	Primary: all-cause mortality at 12 months, patient retention, hospital admission, outpatient attendances, TB, cryptococcal meningitis, ART adherence	Dec 2014
REALITY [28]	Kenya, Malawi, Uganda, Zimbabwe	Individual	CD4 count <100, aged ≥5 years	2x2x2 factorial: a) intensified ART (triple therapy plus raltegravir) for 12 weeks; b) multidrug prophylaxis against co-infections (isoniazid, pyridoxine, co-trimoxazole and fluconazole for 12 weeks; azithromycin for 5 days; single dose albendazole); c) ready-to-use supplementary food for 12 weeks. Comparator: standard of care including co-trimoxazole, with isoniazid and pyridoxine after 12 weeks	Primary: mortality over the first 24 weeks after starting ART, mortality at 48 weeks after starting ART, safety, endpoints relating to the specific mechanisms of action of each intervention	Aug 2015
STATIS [26]	Cambodia, Côte d’Ivoire, Uganda, Vietnam	Individual	CD4 count <100; starting ART; aged ≥18 years. Excluded if overt evidence of TB	Empirical treatment. Comparator: extensive TB screening (point of care urine lipoarabinomannan, sputum Xpert MTB/RIF, chest radiograph for all at enrolment and those with TB symptoms or signs at all follow-up visits)	Primary: composite of (i) 24-week all-cause mortality and (ii) 24-week incidence of invasive bacterial infections; incidence of TB; safety	Jun 2017
TB Fast Track [23]	South Africa	Clinic	CD4 count ≤150; not on ART and willing to start ART; aged ≥18 years. No pre-screening for TB prior to enrolment	Management strategy to identify those at highest risk of TB, so that they can start TB treatment immediately, followed by ART. Comparator: standard of care	Primary: 6-month mortality, severe morbidity over 6 months, time to initiation of ART, retention in ART care, safety	Dec 2015

^a^Terminated early due to insufficient enrolment. All CD4 counts are measured in cells/μL ART antiretroviral therapy, TB tuberculosis.

Other studies are testing alternative interventions designed to reduce early mortality during ART. The REMSTART study included patients with a CD4 count under 200 cells/μL in Tanzania and Zambia, who were individually randomised to standard of care or a complex intervention comprising immediate ART start, screening for cryptococcal antigen, weekly home visits from lay workers and screening with Xpert MTB/RIF at around six weeks [27]. Recruitment to REMSTART is complete. The REALITY trial is recruiting ART-naïve adults and children over five years with a CD4 count under 100 cells/μL in Kenya, Malawi, Uganda and Zimbabwe [28]. There are three interventions: a) intensification of ART using raltegravir in addition to a standard three-drug ART regimen, compared to standard ART; b) multi-drug prophylaxis against co-infections using isoniazid, pyridoxine and co-trimoxazole plus fluconazole for 12 weeks, azithromycin for five days and single-dose albendazole, compared to co-trimoxazole alone for 12 weeks followed by isoniazid and pyridoxine and c) food supplementation compared to standard of care.

We planned the TB Fast Track trial to be as pragmatic (rather than explanatory) as possible, because we wanted to know if the intervention would improve patient outcomes under routine conditions in resource-limited settings. Assessing against criteria set out by Thorpe *et al*. [29], features of the trial consistent with the pragmatic end of this spectrum include few exclusion criteria; an intervention delivered by nurses, as it would be if the intervention was implemented in practice; a routine practice comparator, with few study activities in the control arm likely to alter the standard of care; few study follow-up visits; a primary outcome, all-cause mortality, which is objective and clinically meaningful; no special measures to promote participant adherence and an intention-to-treat primary analysis.

In a few domains, the trial is less pragmatic: the initial study intervention (implementation of a management algorithm) is relatively inflexible and undertaken by study staff, although after the initial management pathway has been determined (treatment for tuberculosis followed by ART, or ART alone), further management is by clinic staff following their usual procedures.

In line with this pragmatic approach, we tried to minimise (in both arms of the study) additional tests for tuberculosis which are not currently routine practice in primary care clinics, which would make the intervention very difficult to replicate in resource-constrained settings. The exception to this principle was that we requested a sputum specimen, processed for smear and culture in a research laboratory, from all participants in the intervention arm in order to have a gold standard against which to assess our management algorithm. These results are delivered to clinic staff as soon as they become available. In our experience to date, they rarely change the patient’s management since most patients are sputum-smear negative, and by the time the culture result is available, most patients have already started tuberculosis treatment. The disadvantage of this minimalist approach to additional diagnostic tests is that we do not have a rigorous gold standard against which to assess the performance of our algorithm. In addition, if the trial succeeds in reducing mortality, it may be difficult to assess the relative contributions of prompt tuberculosis treatment and earlier initiation of ART.

A potential challenge to the TB Fast Track trial is the evolution of South African guidelines for the management of HIV-positive patients. The trial was designed and awarded funding prior to the roll-out of Xpert MTB/RIF in South Africa. When the trial started, no trial clinics had access to Xpert MTB/RIF, but by April 2013 all trial clinic guidelines recommended Xpert MTB/RIF as the first test for people being investigated for tuberculosis. If this guideline was followed rigorously, it could potentially increase detection of active tuberculosis in all study clinics, which could reduce the effect of the intervention. In addition, guidance concerning the initiation of ART has been modified in South Africa, such that several provinces recommend immediate (same day) ART initiation among HIV-positive people who are not taking ART and who have CD4 counts below 200 cells/μL. The guidance does not make clear how screening for active tuberculosis should fit into the process of rapid ART initiation. Rigorous implementation of this guidance would result in individuals in both arms of the study starting ART more rapidly than previously, and thus has the potential to reduce the effect of the intervention. On the other hand, all changes in routine practice which are likely to reduce early mortality are important, and the trial is most useful if it evaluates the intervention against the contemporary standard of care.

This trial will test whether a management algorithm, using tests which can be performed in primary care settings with very few resources, will enable nurses to identify HIV-positive patients at the highest risk of tuberculosis to facilitate prompt treatment, and thus reduce early mortality. The intervention will inevitably result in over-treatment for tuberculosis. We believe this can be justified in the current context where diagnostic tests for tuberculosis lack sensitivity amongst the highest risk patients, and there is consistent evidence that untreated tuberculosis is the most important cause of death in this patient group. There remains an urgent priority for better diagnostic tests for tuberculosis, encompassing those with advanced HIV disease and extrapulmonary tuberculosis, or both, which will render the approach of empirical treatment unnecessary.

## Trial status

The study completed enrolment on 23 December 2014.

## Abbreviations

ART: Antiretroviral therapy
BMI: Body mass index
CXR: Chest radiograph
DALY: Disability-adjusted life year
DMC: Data monitoring committee
LAM: Lipoarabinomannan
TSC: Trial steering committee
